# Burden of Arrhythmias in Epilepsy Patients: A Nationwide Inpatient Analysis of 1.4 Million Hospitalizations in the United States

**DOI:** 10.7759/cureus.1550

**Published:** 2017-08-08

**Authors:** Rupak Desai, Chintan Rupareliya, Upenkumar Patel, Syeda Naqvi, Smit Patel, Abhishek Lunagariya, Zabeen Mahuwala

**Affiliations:** 1 Research Coordinator, Atlanta Veterans Affairs Medical Center; 2 Department of Neurology, University of Missouri, Columbia, Missouri; 3 Public Health, National University; 4 Jinnah Postgraduate Medical Centre, Jinnah Sindh Medical University (SMC); 5 Department of Neurology, University of Connecticut Health Center; 6 Department of Neurology, University of California San Diego; 7 Department of Neurology, University Of Kentucky College of Medicine

**Keywords:** in-hospital outcomes, epilepsy, seizures, sudep, arrhythmias, dysautonomia

## Abstract

Arrhythmias have been one of the common complications in epilepsy patients and have also been the reason for death. However, limited data exist about the burden and outcomes of arrhythmias by subtypes in epilepsy. Our study aims at evaluating the burden and differences in outcomes of various subtypes of arrhythmias in epilepsy patient population. The Nationwide Inpatient Sample (NIS) database from 2014 was examined for epilepsy and arrhythmias related discharges using appropriate International Classification of Disease, Ninth Revision Clinical Modification (ICD-9-CM) codes. The frequency of arrhythmias, gender differences in arrhythmia by subtypes, in-hospital outcomes and mortality predictors was analyzed. A total of 1,424,320 weighted epilepsy patients was determined and included in this study. Around 23.9% (n =277,230) patients had cardiac arrhythmias. The most frequent arrhythmias in the descending frequency were: atrial fibrillation (AFib) 9.7%, other unspecified causes 7.3%, sudden cardiac arrest (SCA) 1.4%, bundle branch block (BBB) 1.2%, ventricular tachycardia (VT) 1%. Males were more predisposed to cardiac arrhythmias compared to females (OR [odds ratio]: 1.1, p <0.001). The prevalence of most subtypes arrhythmias was higher in males. Arrhythmias were present in nearly a quarter of patients with epilepsy. Life threatening arrhythmias were more common in male patients. The length of stay (LOS) and mortality were significantly higher in epilepsy patients with arrhythmia. It is imperative to develop early diagnosis and prompt therapeutic measures to reduce this burden and poor outcomes due to concomitant arrhythmias in epilepsy patients.

## Introduction

The heart and brain are two vital organs for life to be under control of an autonomic nervous system and there is a coordination between them. Any pathological conditions involving one of them might influence the other. We aimed at studying the presence of arrhythmia in epileptic patients. In this study, we also analysed about the frequency and overall incidence of various subtypes of arrhythmias including atrial fibrillation (AFib), atrial flutter (AFL), ventricular fibrillation (V-fib), ventricular flutter (VFL), sinoatrial (SA) dysfunction, atrioventricular (AV) block, premature atrial complex (PAC), premature ventricular contractions (PVC), paroxysmal supraventricular tachycardia (PSVT), bundle branch block (BBB), Wolff Parkinson White syndrome (WPW) and sudden cardiac arrest (SCA). Autonomic dysfunction in epilepsy is one of the causes of cardio respiratory abnormalities in patients suffering from epilepsy. Those cardiorespiratory abnormalities might cause apnea, arrhythmia and sudden death in patients [1-2].

Many authors believed that the cause of sudden unexpected death in epilepsy (SUDEP) is an arrhythmia. Heart rate variability is defined as a marker for autonomic dysfunction, which can eventually lead to sudden death from arrhythmia. Such phenomenon is also seen in Parkinson’s disease and multiple system atrophy. Therefore, heart rate is also an important marker to prevent serious cardiac morbidity in epilepsy. Such variations should not be neglected as it can have hazardous consequences [3-4].

In epilepsy, 10-40% patients suffer intractable seizures, which might lead to sudden death due to cardiorespiratory failure. This study signifies the importance of electrophysiological monitoring in epilepsy patients. The arrhythmia occurs because of the disorder of bioelectrical activity due to the molecular mechanism or as an adverse effect of treatment with antiepileptic drugs (AED) itself. Therefore, an importance of the treatment induced arrhythmia in epilepsy should be kept in mind. Many drugs like carbamazepine, levetiracetam, etc. are notorious for causing cardiac arrhythmias in epilepsy patients [5-6]. 

## Materials and methods

Source of data

We utilized the discharge data from the National Inpatient Sample (NIS) of Healthcare Cost and Utilization Project (HCUP). The NIS is the largest all-payers' data set for inpatient admissions and discharges from almost 1050 United States of America hospitals [7]. It provides cross-sectional data on almost eight million inpatient admissions and discharge data per year and represents around 20% (hospitals from 45 states) stratified sample of all nonfederal hospitals. Data for each hospital admission contains one primary discharge diagnosis and up to 29 secondary diagnoses. The NIS data set does not include long-term care and rehabilitation facilities. The NIS data set is unweighted and it results in the weighted estimate of the total discharge number of the US population when the discharge weight (DISCWT) is applied to the unweighted data. We excluded the data of missing information such as age, gender, discharge condition or primary diagnosis. The NIS is an all payer database of HCUP sponsored by Agency for Healthcare Research and Quality (AHRQ), and it does not require an approval from institutional review board (IRB) because the data set is de-identified. More details on the dataset content and methods of collections are accessible on the HCUP website [7].

The Clinical Classifications Software (CCS) code 83 was used to identify the primary diagnosis of epilepsy shown in Table 1. These codes are used to get the national estimates on various variables in previous studies. Other demographic information that could potentially affect the hospitalization outcomes like obesity, hypertension, diabetes, hyperlipidemia, sepsis, pneumonia and end stage renal disease, etc. were identified using International Classification of Disease, Ninth Edition, Clinical Modification (ICD-9-CM) codes (Table 1).

**Table 1:** International Classification of Disease, Ninth Clinical Modification (ICD-9-CM) and Clinical Classifications Software (CCS) codes used to identify subtypes of arrhythmias and epilepsy. Abbreviations: ICD-9= International Classification of Disease, Ninth Revision, CCS= Clinical Classifications Software.

Condition	Source	Codes
Atrial fibrillation (AF)	ICD-9	427.31
Atrial flutter (Afl)	ICD-9	427.32
Wolff-Parkinson-White syndrome (WPW)	ICD-9	426.7
Nonparoxysmal atrioventricular (AV) nodal tachycardia	ICD-9	426.89
Paroxysmal supraventricular tachycardia (PSVT)	ICD-9	427.0
Paroxysmal ventricular tachycardia (VT)	ICD-9	427.1
Ventricular premature beats	ICD-9	427.69
Ventricular fibrillation (VF)	ICD-9	427.41
Ventricular flutter (Vfl)	ICD-9	427.42
Sudden Cardiac Arrest (SCA)	ICD-9	427.5
Premature atrial/ventricular complexes (PAC/PVC)	ICD-9	427.60, 427.61, 427.69
Sinoatrial (SA) node dysfunction	ICD-9	427.81
Bundle branch block (BBB)	ICD-9	426.2, 426.3, 426.4, 426.50, 426.51, 426.52, 426.53, 426.54
Atrioventricular (AV) blocks	ICD-9	426.0, 426.10, 426.11, 426.12, 426.13
Other, unspecified	ICD-9	427.2, 427.9, 427.89, 785.0
Epilepsy	CCS	83

Study outcomes and variables

We evaluated the frequency of various subtypes of arrhythmias in epilepsy patients. Subtypes of arrhythmia were identified using ICD-9-CM codes shown in Table 1. In order to assess the outcome of hospitalization owing to arrhythmias among epilepsy patient population, the length of stay (LOS), in-hospital mortality and total charges were used. 

Statistical analysis

We used the Statistical Package for the Social Science, version 22.0 from (IBM Corp., Armonk, NY, USA) for all the statistical analysis. To generate the national estimate, we applied weights to unweighted records obtained from NIS. We compared demographics and comorbid risk factors with arrhythmias and without arrhythmias in the patients hospitalized with the primary diagnosis of epilepsy. Pearson’s chi-square test was used for categorical data and the independent sample T-test was used for continuous data. Multivariable logistic regression was used to assess the impact of arrhythmia by subtypes in epilepsy patients after adjusting for variables significant in the univariate analysis. Standard weights provided by HCUP were utilized to get the national weighted estimates of inpatient admissions and p <0.05 was defined as the statistical significance.

## Results

Baseline characteristics

After following rigorous inclusion and exclusion criteria and applying weights for unweighted NIS data set, we could identify 1,424,320 inpatient admissions with the discharge diagnosis of epilepsy in the year 2014. The comparison of baseline characteristics of epilepsy patients with arrhythmias and without arrhythmias is presented in Table 2. Percentages of arrhythmias were the highest (40.8%, p <0.001) in the age group of 65-84 years. Males (51.7% vs. 48.3%, p <0.001) were more prone to have arrhythmias than females. Percentages of Caucasians were higher in arrhythmia group (69.0% vs. 64.0%, p <0.001). Discharge to either skilled nursing facility or intermediate care facility was higher in arrhythmia cohort (33.8% vs. 21.2%, p <0.001).

**Table 2:** Baseline characteristics of hospitalized epilepsy patients without versus with arrhythmia and epilepsy. *Significant P-values ≤ 0.05 at 95% confidence Interval Abbreviations: SNF- Skilled Nursing Facility, INF- Intermediate Nursing Facility, AMA- Against Medical Advice, HMO- Health Maintenance Organization.

Variables	No Arrhythmia	Arrhythmia	P-value*
Unweighted admissions	229418	55446	
Weighted admissions	1147090	277230	
Age in years at admission			
< 18	10.6%	4.1%	<0.001
18 to 44	27.2%	12.7%	<0.001
45 to 64	36.5%	28.6%	<0.001
65 to 84	21.6%	40.8%	<0.001
>85	4.1%	13.8	<0.001
Died during hospitalization			
Did not die	98.2%	91.1%	<0.001
Died	1.8%	8.9%	<0.001
Disposition of Patient			
Routine	60.4%	37.7%	<0.001
Transfer to short-term Hospital	2.6%	3.1%	<0.001
Other Transfers (SNF, ICF)	21.2%	33.8%	<0.001
Home Health Care	11.6%	15.2%	<0.001
Against Medical Advice (AMA)	2.4%	1.3%	<0.001
Elective Vs. Non-elective Admissions			
Non-elective	84.5%	90.9%	<0.001
Elective	15.5%	9.1%	<0.001
Indicator of Sex			
Male	48.1%	51.7%	<0.001
Female	51.9%	48.3%	<0.001
Primary Expected Payer			
Medicare	43.6%	64.5%	<0.001
Medicaid	27.8%	15.8%	<0.001
Private including HMO	20.6%	14.6%	<0.001
Self - Pay	4.5%	2.8%	<0.001
Race			
Caucasian	64.0%	69.0%	<0.001
Afro-American	20.6%	18.4%	<0.001
Hispanic	10.1%	7.6%	<0.001
Asian or Pacific Islander	1.4%	1.6%	<0.001
Native American	0.7%	0.6%	<0.001
Other	3.1%	2.8%	<0.001
Bed Size of Hospital			
Small	17.0%	16.9%	<0.001
Medium	27.9%	28.9%	<0.001
Large	55.1%	54.3%	<0.001
Location/Teaching Status of Hospital			
Rural	8.2%	7.8%	<0.001
Urban - non teaching	23.2%	24.8%	<0.001
Urban - teaching	68.6%	67.4%	<0.001

Baseline comorbidities

Table 3 shows a comparison of different comorbidities between arrhythmia and no arrhythmia cohorts. Comorbidities which are higher in non-arrhythmia cohort are depression (15.7% vs. 15.5%, p <0.001), psychosis  (12.1% vs. 9.7%, p<0.001) and alcohol abuse (8.5% vs. 7.2%, p <0.001). While comorbidities that are higher in arrhythmia cohorts are congestive heart failure (18.7% vs. 6.3%, p <0.001), valvular disorders (7.7% vs. 2.4%, p<0.001), paralysis (12.8% vs. 11.3%, p <0.001), coagulopathy (9.5% vs. 6.2%, p <0.001), diabetes (22.4% vs. 16%, p <0.001) and fluid and electrolyte disorders (43.8% vs. 30%, p <0.001). Comparison of other comorbidities is also shown in Table 3.

**Table 3:** Co-morbidities of hospitalized epilepsy patients without versus with arrhythmia. *Significant P-values ≤ 0.05 at 95% confidence Interval, ^#^Variables are Agency for Healthcare Research and Quality (AHRQ) co-morbidity measures Abbreviations: AIDS- acquired immunodeficiency syndrome, RA- rheumatoid arthritis, CVD- collagen vascular disease.

Variables	No Arrhythmia	Arrhythmia	P-value*
Co –morbidities^#^			
AIDS	0.5%	0.3%	<0.001
Musculoskeletal			
RA/CVD	2.8%	3.1%	<0.001
Cardiovascular			
Congestive Heart Failure	6.3%	18.7%	<0.001
Valvular Disease	2.4%	7.7%	<0.001
Peripheral Vascular Disorders	4.0%	8.4%	<0.001
Respiratory			
Chronic Pulmonary Disease	20.7%	25.5%	<0.001
Pulmonary Circulation Disorders	1.5%	4.5%	<0.001
Neurological			
Paralysis	11.3%	12.8%	<0.001
Other Neurological Disorders	67.9%	74.8%	<0.001
Psychiatry			
Depression	15.7%	15.5%	0.024
Psychoses	12.1%	9.7%	<0.001
Alcohol Abuse	8.5%	7.2%	<0.001
Hemato-oncological			
Deficiency Anemia	17.5%	25.0%	<0.001
Chronic Blood Loss Anemia	0.9%	1.1%	<0.001
Coagulopathy	6.2%	9.5%	<0.001
Weight Loss	5.7%	8.7%	<0.001
Metastatic Cancer	1.8%	2.0%	<0.001
Solid Tumor without Metastasis	1.9%	2.2%	<0.001
Lymphoma	0.5%	0.9%	<0.001
Endocrinological			
Diabetes, Uncomplicated	16.0%	22.4%	<0.001
Hypothyroidism	12.1%	16.7%	<0.001
Renal			
Renal Failure	9.8%	18.7%	<0.001
Fluid and Electrolyte Disorders	30.0%	43.8%	<0.001
Gastrointestinal			
Liver Disease	4.3%	4.1%	<0.001
Obesity	9.2%	11.5%	<0.001

Spectrum and burden of arrhythmias

**Figure 1:**
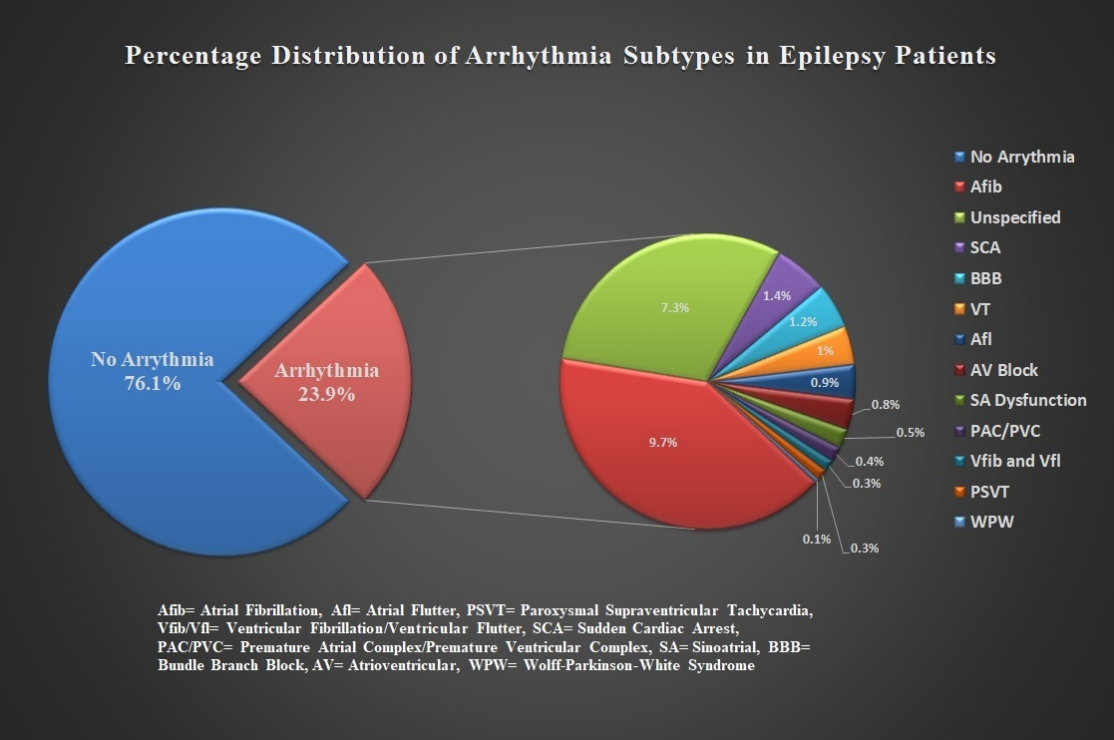
Statistical representation of the percentage distribution of arrhythmias by subtypes in the patients with epilepsy. AFib= atrial fibrillation, Afl= atrial flutter, PSVT= paroxysmal supraventricular tachycardia, Vfib/Vfl= ventricular fibrillation/ventricular flutter, SCA= sudden cardiac arrest, PAC/PVC= premature atrial complex/premature ventricular complex, SA= sinoatrial, BBB= bundle branch block, AV= atrioventricular, WPW= Wolff-Parkinson-white.

A total of 1,424,320 weighted epilepsy patients was included in the study and 23.9% (n =277,230) of patients had cardiac arrhythmias. The most frequent arrhythmias in the descending frequency were: atrial fibrillation (AFib) 9.7%, other unspecified causes 7.3%, bundle branch block (BBB) 1.2%, ventricular tachycardia (VT) 1%, atrial flutter (AFL) 0.9%, atrioventricular (AV) block 0.8% and sinoatrial (SA) node dysfunction 0.5%. Approximately 1.4% patients had sudden cardiac arrest during the hospitalization. Terminal point rhythm was not available for such patients to further categorize the subtype. The burden of other subtypes of arrhythmia in epilepsy patients is shown in detail in Figure 1. Many arrhythmia subtypes have been associated with epilepsy patients [3, 8-9].

Gender differences and in-hospital outcomes

**Figure 2:**
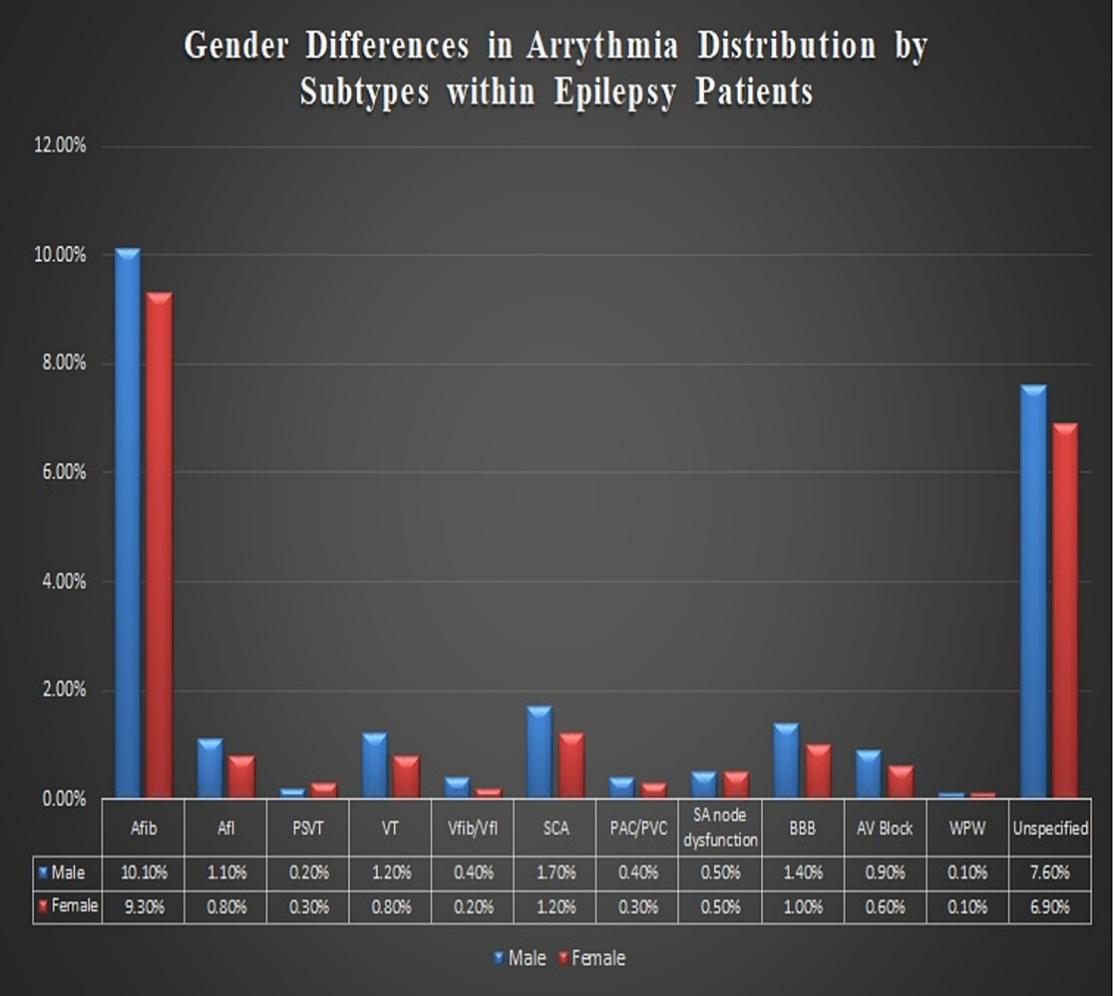
Statistical representation showing the gender differences in arrhythmia distribution within epilepsy patients. AFib= atrial fibrillation, Afl= atrial flutter, PSVT= paroxysmal supraventricular tachycardia, Vfib/Vfl= ventricular fibrillation/ventricular flutter, SCA= sudden cardiac arrest, PAC/PVC= premature atrial complex/premature ventricular complex, SA= sinoatrial, BBB= bundle branch block, AV= atrioventricular, WPW= Wolff-Parkinson-white.

We further analyzed gender differences in the arrhythmia distribution within epilepsy patients. Males were more predisposed to cardiac arrhythmias compared to females (OR: 1.1, p <0.001). Most subtypes of arrhythmias were higher in males (p <0.001). In contrast, paroxysmal supraventricular tachycardia was comparatively higher in females (OR: 1.2, 1.08-1.23, p <0.001)  (Figure 2).

To evaluate the financial burden, we analyzed the in-hospital mortality, the length of stay (LOS) and average total charge of hospitalization incurred due to arrhythmias in epilepsy patients. The findings are shown in Table 4. Mortality rates owing to arrhythmias in epilepsy patients were significantly higher (8.9% vs. 1.8%, p <0.001). The LOS beyond one week was increased in arrhythmia group (p <0.001).

**Table 4:** Association between in-hospital outcomes and arrhythmia in epilepsy patients. *Significant P-values ≤ 0.05 at 95% confidence interval.

Outcome Variables	No Arrhythmia	Arrhythmia	P-value*
Died during hospitalization			
Did not die	98.2%	91.1%	<0.001
Died	1.8%	8.9%	<0.001
Length of stay (cleaned)			
0 to 3 days	50.5%	36.7%	<0.001
4 to 6 days	26.0%	27.4%	<0.001
7 to 9 days	10.5%	14.1%	<0.001
10 to 12 days	4.8%	7.3%	<0.001
>13 days	8.3%	14.5%	<0.001
Total charges (Mean) (USD)	47019.72	75925.28	<0.001

Mortality odds in epilepsy patients with arrhythmia

Table 4 shows the mortality odds associated with different variables in patients of epilepsy with arrhythmia. Top five comorbidity predictors with higher odds of morality are: 1) metastatic cancer (OR= 2.137, 95% CI= 1.983-2.304, p <0.001), 2) coagulopathy (OR= 1.888, 95% CI= 1.815-1.964, p <0.001), 3) pulmonary circulation disorders (OR= 1.603, 95% CI= 1.511-1.700, p <0.001), 4) solid tumor without metastasis (OR= 1.551, 95% CI= 1.432-1.681, p <0.001) and 5) weight loss (OR= 1.344, 95% CI= 1.286-1.405, p <0.001). Associations with other variables are shown in Table 5.

**Table 5:** Multivariate predictors of mortality in epilepsy patients with arrhythmia. *Significant P-value ≤ 0.05 at 95% confidence interval ^#^Variables are Agency for Healthcare Research and Quality (AHRQ) co-morbidity measures Abbreviations: RA- rheumatoid arthritis, CVD- collagen vascular disease, CI - confidence interval.

Variables	Odds Ratio	95% CI	P-value*
Age in years at admission			
<18	Referent	Referent	
18-44	1.219	1.107-1.343	<0.001
45-64	1.606	1.464-1.762	<0.001
65-84	1.685	1.536-1.848	<0.001
>85	1.798	1.631-1.982	<0.001
Elective Vs. Non-elective Admissions			
Non-elective	1.440	1.358-1.528	<0.001
Elective	Referent	Referent	
Indicator of Sex			
Male	Referent	Referent	
Female	1.068	1.037-1.099	<0.001
Length of stay (cleaned)			
1 to 3 days [1]	1.503	1.420-1.592	<0.001
4 to 6 days [2]	0.913	0.860 - 0.969	0.003
7 to 9 days	Referent	Referent	
10 to 12 days [4]	1.050	0.985-1.119	0.136
≥13 days [5]	1.067	1.003-1.135	0.003
Race			
Caucasian	Referent	Referent	
Afro-American	0.929	0.894-0.966	<0.001
Hispanic	1.124	1.065-1.186	<0.001
Asian or Pacific Islander	1.567	1.425-1.723	<0.001
Native American	1.250	1.058-1.476	0.009
Other	1.477	1.367-1.595	<0.001
Co –morbidities^#^			
RA/CVD	.849	0.778-0.925	<0.001
Congestive Heart Failure	1.161	1.119-1.203	<0.001
Peripheral vascular disorders	1.091	1.039-1.145	<0.001
Hypertension	.876	0.849 - 0.904	<0.001
Chronic pulmonary disease	0.960	0.929 – 0.993	<0.001
Pulmonary Circulation Disorders	1.603	1.511-1.700	<0.001
Paralysis	0.787	0.751-0.824	<0.001
Other neurological disorders	1.314	1.268-1.361	<0.001
Depression	0.572	0.546 - 0.600	<0.001
Deficiency anemia	.801	0.774-0.828	<0.001
Coagulopathy	1.888	1.815 - 1.964	<0.001
Weight Loss	1.344	1.286- 1.405	<0.001
Metastatic cancer	2.137	1.983-2.304	<0.001
Solid Tumor without Metastasis	1.551	1.432-1.681	<0.001
Diabetes, uncomplicated	1.170	1.130 - 1.211	<0.001
Hypothyroidism	0.702	0.673- 0.732	<0.001
Renal failure	1.437	1.387-1.789	<0.001
Fluid and Electrolyte Disorders	2.238	2.172-2.306	<0.001
Liver Disease	1.114	1.043-1.189	<0.001

## Discussion

In this study, we tried to determine the different subtypes of arrhythmia in epilepsy. There was an increased incidence observed in the age group of 65-84 years. Most cases were observed in Caucasians. On the basis of comorbidities, the significant difference was seen in the presence of cardiovascular, pulmonary, neurological, iron deficiency, renal dysfunction and diabetes comorbid population. Fluid and electrolyte imbalance played a vital role in cardiac arrhythmia in these patients. The most common subtype observed was atrial fibrillation. A scarce literature is present about the subtypes of arrhythmia, but most of the authors agreed on the point that it is a very common cause of sudden death in epilepsy. In our practice, crucial importance is given to the management of the seizure episode during an emergency. Along with the treatment, monitoring the patient for arrhythmia is also of significant importance [10-11].

The incidence of arrhythmia in epilepsy can be due to a causal association, shared risk factor and resulting from epilepsy treatment itself. Most common of them is ictal asystole. Others being postictal asystole, ictal bradycardia, ictal atrioventricular (AV) conduction block, postictal AV-conduction block, postictal atrial flutter/atrial fibrillation and postictal ventricular fibrillation [12-13]. The patients with chronic epilepsy or drug resistant epilepsy may present with abnormalities of both sinoatrial (SA) and ventricular arrhythmia. These arrhythmias can be detrimental, leading to SUDEP. There are many mechanisms proposed including activation or inhibition of cortical autonomic centers, increase in vagal tone through activation of brainstem reflex centers and respiratory failure. Although the exact pathophysiology is not known [14-15].

Growing up with epilepsy is socially stigmatized. As the age progresses, the comorbidities also increase. The essential rationale for writing this manuscript is to increase an awareness among physicians to improve the quality of life and prevent as many complications as we can. According to American Epilepsy Society (AES), the most common cause of mortality associated with this disease is SUDEP, excluding status epilepticus and other etiologies [16- 17]. The SUDEP affects around 5000 patients only in the United States. The incidence is higher in patients with generalized tonic clonic seizures (GTCS). It is also encouraged that the best way to prevent SUDEP is treating GTCS [16, 18].

The major mechanisms behind SUDEP are cardiac arrhythmias and asphyxia. In the case of arrhythmia, the exact etiology is still unknown. Authors have suggested different theories like inflammation by Interleukin-6 (IL-6), autonomic dysregulations or altered ion channel due to drug resistant epilepsy [2, 4, 19]. The patients suffering from temporal lobe epilepsy are seen to be at increased risk of post-ictal arrhythmia due to autonomic instability. Physicians and nurses in the emergency department should be well trained for this dreadful complication in a patient presenting with seizure episode [13, 20].

Limitations

This study carries few important limitations. First, all the patients with the diagnosis of epilepsy were included in the study and further stratification in the context of various subtypes of epilepsy could not be performed because of limitations of CCS coding. Likewise, the substantial portion of arrhythmia subtypes must be included under the category of unspecified causes due to coding limitations. Second, NIS database does not have the information about electroencephalogram (EEG) parameters for the specific subtype of epilepsy and it could not be found which type of epilepsy, whether generalized or localized, had the highest correlation with arrhythmias. The NIS database contains a discharge-based data; same patients who might have been admitted previously with different diagnosis might have been included again. Nonetheless, this is the first study, which systematically analyzes burden of arrhythmias by subtypes in the large sample of epilepsy population. Further research is suggested in this direction, focusing on finding a relationship between epilepsy and arrhythmias, using prospective randomized trials. 

## Conclusions

In a nutshell, arrhythmias are frequent in 24.16% of population hospitalized with epilepsy with atrial fibrillation being the most common subtype. Except for PSVT, all the other subtypes showed significant prevalence among males. Mortality owing to arrhythmias in epilepsy patients was higher. It also led to greater financial burden and utilization of other healthcare resources.
